# IFI16 Inhibits Porcine Reproductive and Respiratory Syndrome Virus 2 Replication in a MAVS-Dependent Manner in MARC-145 Cells

**DOI:** 10.3390/v11121160

**Published:** 2019-12-16

**Authors:** Xiaobo Chang, Xibao Shi, Xiaozhuan Zhang, Li Wang, Xuewu Li, Aiping Wang, Ruiguang Deng, Enmin Zhou, Gaiping Zhang

**Affiliations:** 1 College of Veterinary Medicine, Northwest A&F University, Yangling 712100, China; 2 Henan Provincial Key Laboratory of Animal Immunology, Henan Academy of Agricultural Sciences, Zhengzhou 450002, China; 3 College of Life Sciences, Henan Normal University, Xinxiang 453007, China; 4 Department of Bioengineering, Zhengzhou University, Zhengzhou 450000, China; 5 College of Animal Science and Veterinary Medicine, Henan Agricultural University, Zhengzhou 450002, China; 6 Jiangsu Co-Innovation Center for Prevention and Control of Important Animal Infectious Diseases and Zoonoses, Yangzhou 225009, China

**Keywords:** PRRSV, IFI16, antiviral protein, MAVS

## Abstract

Porcine reproductive and respiratory syndrome virus (PRRSV) is a single-stranded positive-sense RNA virus, and the current strategies for controlling PRRSV are limited. Interferon gamma-inducible protein 16 (IFI16) has been reported to have a broader role in the regulation of the type I interferons (IFNs) response to RNA and DNA viruses. However, the function of IFI16 in PRRSV infection is unclear. Here, we revealed that IFI16 acts as a novel antiviral protein against PRRSV-2. IFI16 could be induced by interferon-beta (IFN-β). Overexpression of IFI16 could significantly suppress PRRSV-2 replication, and silencing the expression of endogenous IFI16 by small interfering RNAs led to the promotion of PRRSV-2 replication in MARC-145 cells. Additionally, IFI16 could promote mitochondrial antiviral signaling protein (MAVS)-mediated production of type I interferon and interact with MAVS. More importantly, IFI16 exerted anti-PRRSV effects in a MAVS-dependent manner. In conclusion, our data demonstrated that IFI16 has an inhibitory effect on PRRSV-2, and these findings contribute to understanding the role of cellular proteins in regulating PRRSV replication and may have implications for the future antiviral strategies.

## 1. Introduction

Porcine reproductive and respiratory syndrome (PRRS), caused by PRRS virus (PRRSV), is an economically important viral disease all over the word [1,2]. PRRSV is a member of the *Arteriviridae* family in the order *Nidovirales* and consists of an enveloped 15 kb positive-strand RNA genome containing at least ten open reading frames (ORFs) [3,4,5,6]. PRRSV is divided into two genotypes: the European genotype (type 1) and the North American genotype (type 2). There is considerable sequence variability within both groups, and only about 50–60% nucleotide sequence identity between the two subtypes [7,8]. Recently, based on a new proposed classification scheme, type 1 and type 2 PRRSV have been classified into two species and renamed PRRSV-1 and PRRSV-2, respectively [9,10]. PRRSV has genetic diversity and has evolved multiple mechanisms to evade the host immune response [11,12]. Currently, there are no effective control strategies against PRRS.

Type I interferons (IFN-α/β) play pivotal roles in the innate defense against viral infection. During virus infection, viral nucleic acids are the main pathogen-associated molecular patterns (PAMPs) which can be detected by the cellular receptors such as retinoic acid-inducible gene I (RIG-I)-like receptors (RLRs) and interferon gamma-inducible protein 16 (IFI16) (DNA sensor). Then, RIG-I recruits the mitochondrial antiviral signaling (MAVS), while IFI16 activates the endoplasmic reticulum signaling adaptor STING, leading to TANK-binding kinase 1 (TBK1)-dependent phosphorylation of interferon regulatory factor 3 (IRF3) and transcription of type I interferons (IFNs) [13,14,15,16]. Then, type I interferons bind to IFN-α/β receptors and induce the production of a large number of interferon-stimulated genes (ISGs) in response to virus [17].

Previous studies have demonstrated that IFN-α, IFN-β, and IFN-γ have an antiviral effect against PRRSV-2 [18,19,20,21,22]. In addition, interferon-stimulated genes (such as *OAS1* and *Mx2*) have been confirmed to have antiviral effects against type 2 PRRSV strain S1 and PRRSV-WUH3 strain [23,24], which indicated that interferon-stimulated genes play important roles in inhibiting PRRSV replication. IFI16, a member of the PYHIN protein family that contains a pyrin domain and two DNA-binding HIN domains, displays multifaceted activity due to its ability to bind to various target proteins and, in turn, modulate various functions including direct actions in regulation of transcription, proliferation, differentiation, apoptosis, antiviral restriction, and inflammation [25,26,27,28]. Besides, IFI16 has been reported to have a broader role in the regulation of interferon-stimulated genes expression, leading to responses to not only DNA viruses, but also RNA viruses, such as Sendai virus [29,30]. However, the role of IFI16 in regulating PRRSV replication is unclear. So, this study aimed to explore whether IFI16 could inhibit PRRSV replication.

In this study, we have shown that IFI16 could suppress PRRSV-2 replication for the first time. Besides, IFI16 could positively regulate MAVS-mediated type I IFN signaling pathway and interact with MAVS. Furthermore, MAVS was essential for IFI16 to inhibit PRRSV-2 replication, which suggested that IFI16 plays an important role in the antiviral response to PRRSV-2, and these findings may have implications for the future control of PRRS.

## 2. Materials and Methods

### 2.1. Cells and Virus

HEK-293T (a human embryonic kidney cell line) and PRRSV permissive MARC-145 (an African green monkey kidney cell line) cells were cultured in Dulbecco’s modified Eagle’s medium (DMEM) (Gibco, Grand Island, NY, USA) supplemented with 10% FBS (Gibco), 100 U/mL penicillin, and 100 µg/mL streptomycin (Gibco) at 37 °C in a humidified atmosphere of 5% CO_2_. PRRSV-2 strain BJ-4 (GenBank No. AF331831) was a kind gift from Prof. Hanchun Yang (China Agricultural University).

### 2.2. Antibodies and Reagents

Mouse anti-Flag M2 monoclonal antibody, mouse anti-c-Myc monoclonal antibody, anti-Flag affinity gel beads, anti-c-Myc affinity gel beads, and mouse IgG agarose were purchased from Sigma-Aldrich. PRRSV N protein antibody was purchased from GeneTex. Mouse anti-β-actin monoclonal antibody was purchased from GenScript. Rabbit anti-MAVS monoclonal antibody was purchased from Cell Signaling Technology (CST). The secondary antibodies conjugated to HRP were purchased from Jackson Immuno Research. Polyinosinic-polycytidylic acid (polyI:C) was purchased from Sigma-Aldrich. Recombinant human IFN-β was purchased from PEPROTECH. Lipofectamine2000 transfection reagent and Lipofectamine RNAiMAX transfection reagent were purchased from Invitrogen (Carlsbad, CA, USA).

### 2.3. Plasmids

To generate IFI16-Myc, the cDNA of IFI16 from MARC-145 cells was amplified and cloned into pCMV-Myc vector (Beyotime, Shanghai, China). The genes of RIG-I, MDA5, MAVS, TBK1, and IRF3 from MARC-145 cells were cloned into pCMV-Flag (Beyotime), creating RIG-I-Flag, MDA5-Flag, MAVS-Flag, TBK1-Flag, and IRF3-Flag respectively. The expression plasmids for 3×Flag-tagged MAVS and MAVS truncations were constructed by standard molecular cloning method from cDNA templates. All constructs were confirmed by DNA sequencing. The IFN-β promoter reporter plasmid (p-284) has been constructed as previous described [31]. All primers used are listed in Table 1.

### 2.4. Gene Silencing with siRNA

The specific siRNAs targeting IFI16, MAVS, and the control siRNA (siNC) were synthesized by GenePharma. The siRNA sequences are as follows: siIFI16, 5′-CCAAGCAGCAGUUGCUUAATT-3′; siMAVS, 5′-GCAUCUCUUCAGUACCCUUTT-3′; the nontargeting control siRNA (siNC), 5′-UUCUCCGAACGUGUCACGUTT-3′. MARC-145 cells were transfected with 50 nM siRNA using Lipofectamine RNAiMAX transfection reagent (Invitrogen, Carlsbad, CA, USA). At 24 h post-transfection (hpt), the transfected cells were infected with PRRSV at a multiplicity of infection (MOI) of 0.1. And then the supernatants and lysates of the cells were collected at 24, 36, and 48 h postinfection (hpi) for further analysis.

### 2.5. Quantitative Real-Time PCR Analysis

Total RNA was extracted from cells using RNeasy Mini Kit (Qiagen), reverse transcription was accomplished with PrimeScript™ RT Master Mix (Perfect Real Time) (Takara, Kyoto, Japan) according to the manufacturer’s protocol. And then the samples were subjected to real-time PCR analysis with specific primers by using a Fast Start Universal SYBR Green Master Kit (Roche, Basel, Switzerland) on a 7500 fast RT-PCR system (Applied Biosystems, Foster City, CA, USA). Relative analysis of gene expression was evaluated using the 2^−ΔΔCT^ method, in which target gene expression was normalized by glyceraldehyde 3-phosphate dehydrogenase (GAPDH) expression. Primers are shown in Table 2.

### 2.6. Dual Luciferase Reporter Assay

MARC-145 cells in 24-well plates were transfected with indicated expression plasmids and IFN-β luciferase reporter plasmids by using Lipofectamine 2000 transfection reagent (Invitrogen). At 36 hpt, the cells were transfected with polyI:C (10 µg/mL) (Sigma-Aldrich, St. Louis, MO, USA) for 12 h, and then the cells were harvested and the luciferase activity was measured by using dual-luciferase reporter assay system (Promega, Madison, WI, USA).

### 2.7. Co-Immunoprecipitation

HEK-293T cells were transfected with IFI16-Myc and MAVS-3×Flag, MAVS truncations plasmids or control vector. At 48 hpt, the cells were lysed in IP-lysis buffer (Sigma-Aldrich) containing protease inhibitor cocktail (Roche) for 1 h at 4 °C. The lysates were centrifuged at 12,000× *g* for 15 min at 4 °C, and the supernatants were pre-cleared with mouse IgG-Agarose (Sigma-Aldrich) at 4 °C for 2 h. Then, the pre-cleared supernatants were incubated with anti-c-Myc affinity gel beads or anti-Flag affinity gel beads (Sigma-Aldrich) for 4 h or overnight at 4 °C. The precipitates were washed five times with TBS buffer and detected by western blotting.

### 2.8. Virus Titration

Virus titers were determined according to a previous report [32]. Briefly, MARC-145 cells, grown in 96-well plates, were infected with ten-fold serial dilution of samples. After 1 h incubation at 37 °C, the supernatants were replaced with fresh DMEM containing 2% FBS. Five days post infection, the cytopathic effect (CPE) characterized by clumping and shrinkage of cells was obviously visible in MARC-145 cells and the viral titers, expressed as 50% tissue culture infective dose (TCID_50_), calculated according to the method of Reed-Muench [33].

### 2.9. Statistical Analysis

Statistical graphs were created with GraphPad Prism software, and all data were analyzed using Student’s *t* tests as the mean values ± the standard deviations (SD) of at least three independent experiments. The asterisks in the figures indicate significant differences (*, *p* < 0.05; **, *p* < 0.01).

## 3. Results

### 3.1. IFI16 Inhibits PRRSV-2 Replication

**Figure 1:**
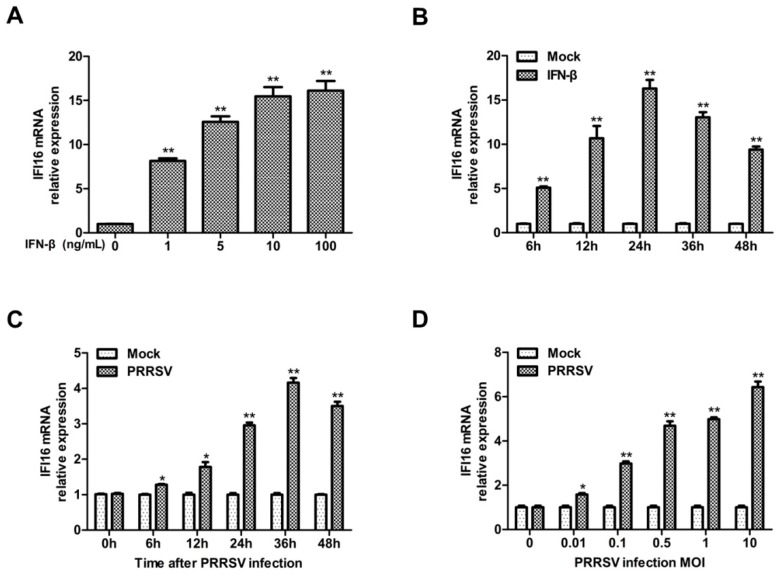
Interferon gamma-inducible protein 16 (IFI16) is upregulated upon interferon-beta (IFN-β) and porcine reproductive and respiratory syndrome virus 2 (PRRSV-2) infection. (**A**) MARC-145 cells were treated with different concentrations of IFN-β, and 24 h later, the mRNA levels of IFI16 were detected by RT-qPCR. (**B**) MARC-145 cells were treated with IFN-β (10 ng/mL) for different times as indicated, and the mRNA levels of IFI16 were examined by RT-qPCR. (**C**) MARC-145 cells were infected with PRRSV-2 BJ-4 at a multiplicity of infection (MOI) of 1, the mRNA levels of IFI16 were examined at 0, 6, 12, 24, 36, and 48 hpi by RT-qPCR. (**D**) MARC-145 cells were infected with PRRSV-2 BJ-4 at different MOIs for 24 h, the mRNA levels of IFI16 were examined by RT-qPCR. All experiments were repeated at least three times with similar results. * *p* < 0.05, ** *p* < 0.01.

Since type I interferon and interferon-induced genes could efficiently inhibit PRRSV replication in MARC-145 cells [21,23], and it has been reported that IFI16 could be induced by type I interferon [34,35,36], we firstly confirmed whether IFI16 could be induced by type I interferon in MARC-145 cells, and then explored whether IFI16 could inhibit PRRSV replication. MARC-145 cells were treated with IFN-β, and then the expression of IFI16 was detected. Consistent with the results of previous reports, IFN-β could also efficiently induce the expression of IFI16 (Figure 1A), and the expression of IFI16 was enhanced in an IFN-β-dose-dependent manner and peaked at 24 h in MARC-145 cells (Figure 1B). In addition, the transcription level of IFI16 was increased in the cells infected with PRRSV-2 (Figure 1C,D).

**Figure 2:**
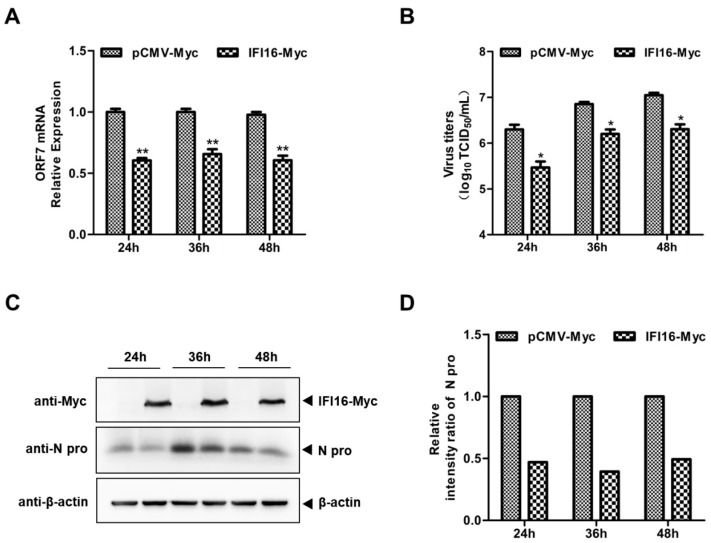
Overexpression of IFI16 inhibits PRRSV-2 replication. MARC-145 cells were transfected with IFI16-Myc or control vector for 24 h, and then infected with PRRSV-2 BJ-4 at a MOI of 0.1 for another 24, 36, and 48 h. (**A**) The RNA levels of PRRSV were assessed by RT-qPCR. (**B**) The virus titers were assayed by TCID_50_ (50% tissue culture infective dose). (**C**) The expression levels of IFI16 and N protein of PRRSV were analyzed by western blotting. (**D**) The relative intensity ratios of N protein were also shown in this figure by using Image J software. All experiments were repeated at least three times with similar results. * *p* < 0.05, ** *p* < 0.01.

**Figure 3:**
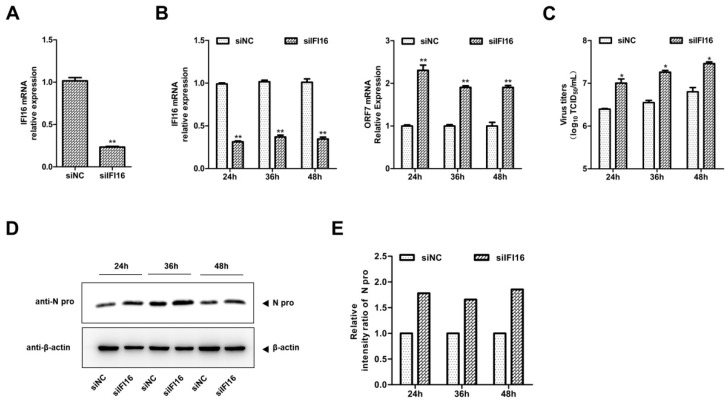
Knockdown of IFI16 enhances PRRSV-2 growth. (**A**) MARC-145 cells were transfected with siIFI16 or a nontargeting control (siNC) for 48 h, the knockdown efficiency of IFI16 was examined by RT-qPCR. (**B**) MARC-145 cells transfected with siIFI16 or siNC were infected with PRRSV-2 BJ-4 at a MOI of 0.1 for 24, 36, 48 h. The mRNA levels of IFI16 and PRRSV ORF7 were assessed by RT-qPCR. (**C**) The virus titers were analyzed by TCID_50_. (**D**) The expression levels of N protein were detected by western blotting with a rabbit anti-PRRSV N protein polyclonal antibody. (**E**) The relative intensity ratios of N protein were analyzed by Image J software. All experiments were repeated at least three times with similar results. * *p* < 0.05, ** *p* < 0.01.

Next, to explore whether IFI16 could inhibit PRRSV-2 replication, MARC-145 cells were transfected with IFI16-Myc and control vector for 24 h, and then the cells were infected with PRRSV-2 at a MOI of 0.1. The results in Figure 2 show that overexpression of IFI16 reduced the RNA levels of PRRSV (Figure 2A), the PRRSV titers (Figure 2B), and the expression levels of the N protein of PRRSV (Figure 2C,D). To further examine the antiviral effects of IFI16 on PRRSV-2, we used specific siRNA to down-regulate the expression of endogenous IFI16, which led to lower expression of IFI16 than that in cells transfected with control siRNA (Figure 3A). Compared with the nontargeting control siRNA (siNC)-transfected control cells, the RNA levels of PRRSV (Figure 3B), viral titers (Figure 3C), and the expression of N protein (Figure 3D,E) were significantly increased in MARC-145 cells transfected with siIFI16. Together, these results indicated that IFI16 acts as an antiviral protein against PRRSV-2.

### 3.2. IFI16 Enhances the MAVS-mediated Type I IFN Signaling

**Figure 4:**
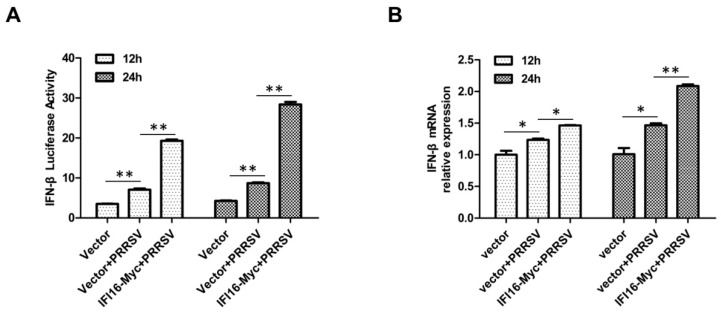
IFI16 enhances PRRSV-induced production of IFN-β. (**A**) MARC-145 cells were transfected with IFI16-Myc or control vector and IFN-β reporter plasmid, and 24 h later, the cells were infected with PRRSV-2 BJ-4 at a MOI of 1 for 12 h or 24 h, and then the luciferase activities were assayed. (**B**) MARC-145 cells were transfected with IFI16-Myc or control vector for 24 h, then infected with PRRSV-2 BJ-4 at a MOI of 1 for 12 h or 24 h, and the mRNA levels of IFN-β were measured by RT-qPCR. All experiments were repeated at least three times with similar results. * *p* < 0.05, ** *p* < 0.01.

IFI16 plays an important role in Sendai virus (SeV)-mediated production of type I IFN [30,37], and in light of the fact that PRRSV could slightly induce the production of type I IFN [31,38], we wonder whether IFI16 was useful to the transcription of type I IFN during PRRSV infection. MARC-145 cells were transfected with IFI16-Myc or control vector, and 24 h later, the cells were infected with PRRSV-2 BJ-4 at a MOI of 1. The results showed that IFI16 could enhance PRRSV-induced production of IFN-β (Figure 4).

**Figure 5:**
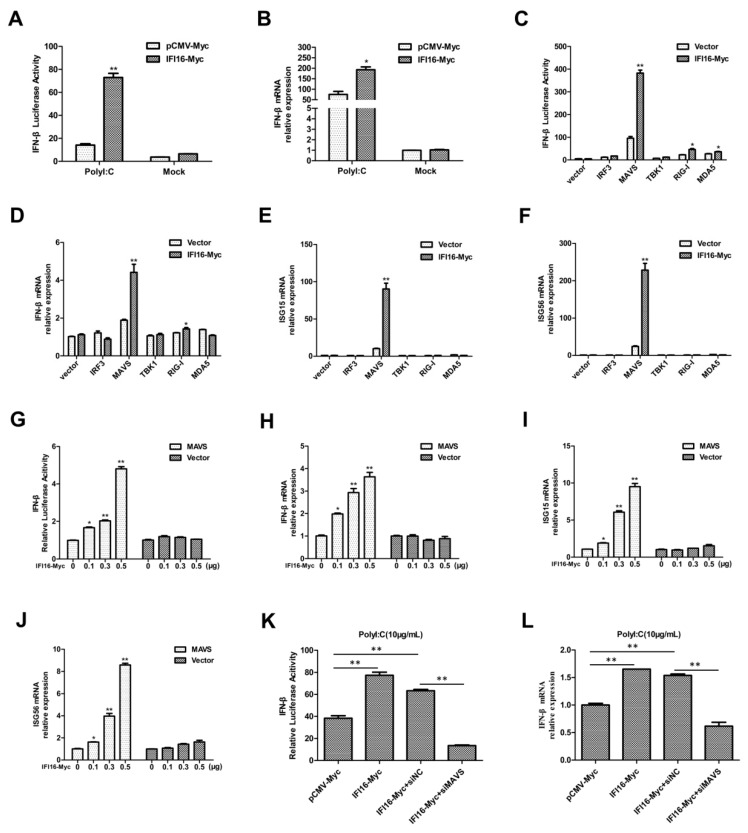
IFI16 enhances MAVS-mediated type I IFN signaling. (**A**) MARC-145 cells were transfected with IFN-β reporter plasmid and IFI16-Myc or control vector, and 36 h later, the cells were transfected with polyI:C (10 µg/mL) for 12 h, and then the luciferase activities were assayed. (**B**) MARC-145 cells were transfected with IFI16-Myc or control vector for 36 h, then transfected with polyI:C for 12 h, the mRNA levels of IFN-β were measured by RT-qPCR. (**C**) MARC-145 cells were transfected with IFN-β reporter plasmid, IFI16-Myc, and IRF3, MAVS, TBK1, RIG-I, or MDA5 plasmids. Luciferase assays were performed at 48 h. (**D**–**F**) MARC-145 cells were transfected with IFI16-Myc and IRF3, MAVS, TBK1, RIG-I, or MDA5 plasmids. The mRNA levels of IFN-β (**D**), ISG15 (**E**), and ISG56 (**F**) were detected by RT-qPCR. (**G**) MARC-145 cells were transfected with IFN-β reporter plasmid, MAVS, and increasing amounts of IFI16-Myc, at 48 hpt, the luciferase activities were analyzed using a dual-luciferase reporter assay. (**H**–**J**) MARC-145 cells were transfected with MAVS and increasing amounts of IFI16-Myc, the mRNA levels of IFN-β (**H**), ISG15 (**I**), and ISG56 (**J**) were detected by RT-qPCR. (**K**) MARC-145 cells were transfected with IFN-β reporter plasmid, IFI16-Myc, and siNC or siMAVS for 36 h, then the cells were stimulated with polyI:C, and 12 h later, the luciferase activities were assayed. (**L**) MARC-145 cells were transfected with IFI16-Myc and siNC or siMAVS for 36 h, and then the cells were stimulated with polyI:C, and 12 h later, the mRNA levels of IFN-β were quantified by RT-qPCR. All experiments were repeated at least three times with similar results. * *p* < 0.05, ** *p* < 0.01.

Given the fact that IFI16 plays a key role in the signaling through RIG-I, and among the different RNA sensors, DExD/H-box RNA helicases of the RLR family have been identified as essential sensors of RNA viruses [15,30,39,40], we explored the role of IFI16 in the RIG-I-mediated signaling pathway. Firstly, we found that IFI16 could enhance polyI:C-induced IFN-β promoter activity and the transcriptional levels of IFN-β (Figure 5A,B). Next, to further investigate the mechanism of IFI16 in enhancing type I IFN signaling, MARC-145 cells were co-transfected with plasmids encoding IFI16, components of RIG-I pathway, and IFN-β reporter plasmid. The results showed that IFI16 greatly enhanced the IFN-β promoter activity, the mRNA transcriptional levels of IFN-β, ISG15, and ISG56 induced by MAVS (Figure 5C–F). Subsequently, cells were co-transfected with MAVS, IFN-β reporter plasmid, and different concentrations of IFI16, and the results showed that IFI16 strongly increased MAVS-mediated IFN-β promoter activity and the mRNA transcription levels of IFN-β, ISG15, and ISG56 in a dose-dependent manner (Figure 5G–J).

Since IFI16 could enhance MAVS-mediated type I IFN signaling, to identify whether MAVS is indispensable for IFI16 to regulate the production of type I IFN, MARC-145 cells were transfected with siMAVS, IFI16-Myc, and IFN-β reporter plasmid, and 36 h later, the cells were stimulated with polyI:C. And the results showed that IFI16 could not promote polyI:C-induced type I IFN production upon silencing of MAVS (Figure 5K,L). Collectively, these data indicated that IFI16 positively regulates MAVS-mediated type I IFN signaling.

### 3.3. IFI16 Interacts with MAVS

**Figure 6:**
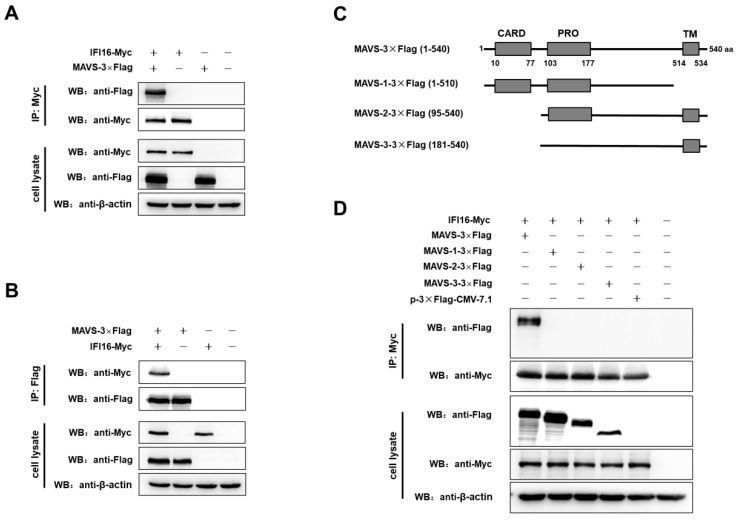
IFI16 interacts with MAVS. (**A**,**B**) HEK-293T cells were co-transfected with IFI16-Myc and MAVS-3×Flag for 48 h. Cells were lysed, and the lysates were subjected to immunoprecipitation and analyzed by western blotting using indicated antibodies. (**C**) Scheme of MAVS protein and its mutants. (**D**) HEK-293T cells were co-transfected with IFI16-Myc and MAVS-3×Flag, MAVS-1-3×Flag, MAVS-2-3×Flag, MAVS-3-3×Flag, p3×Flag-CMV-7.1, and 48 h later, the cells were lysed and subjected to immunoprecipitation and analyzed by western blotting.

IFI16 is mainly localized in nucleus and partially localized in cytoplasmic and mitochondria [29], while MAVS could localize in cytoplasmic and mitochondria [41]. Besides, IFI16 could facilitate the MAVS-mediated type I IFN signaling, so to determine whether IFI16 could interact with MAVS, the co-immunoprecipitation assays (co-IP) were performed in 293T cells, and the results showed that IFI16 specifically interacted with MAVS (Figure 6A,B).

As a central adaptor of IFN signaling, MAVS contains an N-terminal CARD domain, a proline-rich domain, and a C-terminal transmembrane domain (TM) [42,43]. So, to determine the functions of MAVS domains in the binding with IFI16, a series of MAVS truncations were generated (Figure 6C). In the assays of co-IP, IFI16 only interacted with full-length MAVS, but not with truncations of MAVS (Figure 6D). These results indicated that the CARD domain and TM domain of MAVS are sufficient and efficient for the binding with IFI16.

### 3.4. Antiviral Activity of IFI16 is Dependent on MAVS

**Figure 7:**
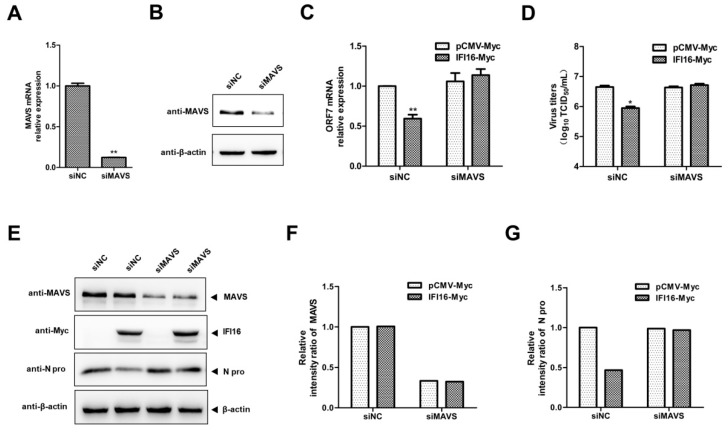
The antiviral activity of IFI16 is dependent on MAVS. (**A**,**B**) MARC-145 cells were transfected with siMAVS or siNC for 48 h, the silence efficiency of MAVS was examined by RT-qPCR and western blotting. (**C**) MARC-145 cells were transfected with siMAVS and IFI16-Myc, and 24 h later, and the cells were infected with PRRSV-2 BJ-4 at a MOI of 0.1 for 24 h. Then, the cells were harvested, and RNA levels of PRRSV were determined by RT-qPCR. (**D**) The virus titers from cell supernatants were analyzed by TCID_50_. (**E**) The expression levels of N protein were detected by western blotting. (**F**,**G**) The relative intensity ratios of MAVS and N protein were analyzed using Image J software. All experiments were repeated at least three times with similar results. * *p* < 0.05, ** *p* < 0.01.

Since IFI16 possesses significant antiviral activity and enhances MAVS-mediated type I IFN signaling, to investigate whether the antiviral activity of IFI16 is dependent on MAVS, we silenced MAVS using siRNAs, and as shown in Figure 7, marked reduction of MAVS expression was observed in the MAVS silenced cells (Figure 7A,B). And, IFI16 could not decrease the PRRSV RNA levels (Figure 7C), PRRSV titers (Figure 7D), and the expression of N protein of PRRSV (Figure 7E–G) in the MAVS-silenced cells. These data indicated that the anti-PRRSV activity of IFI16 needs the involvement of MAVS.

## 4. Discussion

In this study, we revealed a novel mechanism by which IFI16 inhibits PRRSV-2 replication. Initially, we showed that IFI16 could efficiently repress PRRSV-2 replication. Subsequently, we demonstrated that IFI16 markedly enhances MAVS-mediated type I IFN signaling and binds directly with MAVS. Finally, the ability of IFI16 to antagonize the replication of PRRSV-2 is dependent on MAVS. Taken together, these findings suggest that IFI16 plays an important role in response to PRRSV-2 in MARC-145 cells.

IFI16 is an intracellular DNA sensor and could mediate the induction of IFN-β by stimulating with single-stranded and double-stranded DNA sequences or DNA virus infection [29]. And IFI16 has also been described as an antiviral restriction factor against DNA viruses since it could directly interact with DNA virus components and block viral replication [44,45]. Besides, IFI16 plays a broader role in the regulation of the type I IFN response to DNA viruses in antiviral immunity [30]. However, the functions of IFI16 in regulating RNA virus including PRRSV replication are largely unknown. In this study, we provide the first evidence that IFI16 could inhibit PRRSV-2 replication in MARC-145 cells. Additionally, IFI16 is widely expressed in endothelial and epithelial cells [46], and although the roles of IFI16 in porcine alveolar macrophages (PAMs) currently could not be investigated, this study may have implications for other RNA viruses.

It has been reported that IFI16 was essential for SeV-mediated production of type I IFN [30,37], and SeV-mediated induction of type I IFN comes primarily from RIG-I activation [39,40,47], which indicated that IFI16 may be involved in regulating the RIG-I signal pathway. Here, we have shown that IFI16 could interact with MAVS and promote MAVS-mediated type I IFN signaling, and MAVS was essential for IFI16 to regulate type I IFN signaling. MAVS is a central adaptor protein for RIG-I signaling pathway, which indicted that IFI16 may positively regulate RIG-I signaling pathway. Given that IFI16 transcriptionally regulates ISGs to enhance IFN responses to DNA viruses [29,30], this may be a common mechanism in response to DNA or RNA viruses, which needs further demonstration in other PRRSV strains, and even other RNA viruses.

Since PRRSV could be recognized by RIG-I and induce the production of type I IFN [31,38], and IFI16 could positively regulate the RIG-I signaling pathway, we explored the mechanism by which IFI16 inhibits PRRSV-2 replication. We found that IFI16 could facilitate the PRRSV-mediated induction of IFN-β, interact with MAVS, and the anti-PRRSV activity of IFI16 is dependent on MAVS, which indicted that the ability of IFI16 to inhibit PRRSV-2 replication may be relevant to its role in the type I IFN signaling. Interestingly, IFI16 expression was increased after PRRSV infection, while possibly inhibiting virus replication. However, the mechanisms underlying need to be explored further.

In fact, in contrast to the results that IFI16 plays an important role in SeV-mediated production of type I IFN [30], several other groups have shown that both knockout and knockdown of IFI16 could not influence the ployI:C and SeV-mediated production of type I interferon [26,29,48]. The reason of these different results may be due to different cell lines they used. Here, we showed another piece of evidence that IFI16 may take part in the RIG-I signaling pathway. Previous studies have shown that STING could interact with RIG-I and MAVS, and there was also a marked reduction in the induction of IFN-β by RIG-I and MAVS in the absence of STING [49,50], while Brunette et al. have shown that the IFN response to poly(dA:dT) was reduced by >99% in STING^−/−^, MAVS^−/−^ macrophages, and DCs, but not in phagocytes deficient in STING alone [51], which indicated that IFI16 may be a crosstalk between RIG-I-MAVS-type I interferon and STING-DNA-sensing pathways [52]. It is well known that IFI16 recruits STING to induce IFN-β transcription, so the roles of IFI16 in the crosstalk between RIG-I-MAVS-RNA-sensing pathways and IFI16-STING-DNA-sensing pathways need further studies.

In conclusion, our data show for the first time that IFI16 could inhibit PRRSV-2 replication in a MAVS-dependent manner, which may have implications for other RNA viruses and contribute to understand the antiviral mechanism, as well as virus–host interactions.

## Figures and Tables

**Table 1:** Primers used for expression plasmid construction.

Primers	Sequences (5′ to 3′)
IFI16-F	CGCGGATCCATGGAAAAAAAATACAAGAACATTG
IFI16-R	CCGCTCGAGTTAGAAGAAAAAGCCTGGTGAAGTT
RIG-I-F	TCCCCCCGGGCATGACTACGGAGCAGCGGCGCAGCCT
RIG-I-R	ATCTGTCGACTCATTTGGCCATTTCTGCTGGAT
MDA5-F	CGCGGATCCATGTCGAATGGGTATTCCACAGAC
MDA5-R	CCGCTCGAGCTAATCCTCATCACTAAATAAACAGCA
MAVS-F	CCCAAGCTTATGCCGTTTGCTGAAGACAAGACCT
MAVS-R	CCGCTCGAGCTAGTGCAGGCGCCGCCGGTACATC
IRF3-F	CCCAAGCTTATGGGAACCCCAAAGCCACGGAT
IRF3-R	CCGCTCGAGTCAGGTCTCCCCAGGGCCCTGGAA
TBK1-F	CGCGGATCCATGCAGAGCACTTCTAATCATCT
TBK1-R	CCGCTCGAGCTAAAGACAGTCAACGTTGCGA
MAVS-1-F	CCCAAGCTTATGCCGTTTGCTGAAGACAAGACCT
MAVS-1-R	CGGGGTACCCTAGTGGCATGGCCCCTCCCTCT
MAVS-2-F	CCCAAGCTTATGTACCAGCCTCGGACCTCGGAC
MAVS-2-R	CGGGGTACCCTAGTGCAGGCGCCGCCGGTACATC
MAVS-3-F	CCCAAGCTTATGTCCTCTGACCTGGCAGCCCTC
MAVS-3-R	CGGGGTACCCTAGTGCAGGCGCCGCCGGTACATC

**Table 2:** Primers used for qPCR.

Primers	Sequences (5′ to 3′)	Amplicon Size	Final Concentration
ORF7-F	AAACCAGTCCAGAGGCAAGG	221	0.3 µM
ORF7-R	GCAAACTAAACTCCACAGTGTAA		
IFI16-F	TGTGAATGGGGTGTTTGAGGT	169	0.3 µM
IFI16-R	CGGTGCCAATTCAAAGCAGG		
IFN-β-F	ACGGCTCTTTCCATGAGCTAC	185	0.3 µM
IFN-β-R	GTCAATGCAGCGTCCTCCTT		
ISG15-F	CTGAAGGCAAAGATCGCCCA	186	0.3 µM
ISG15-R	GTCGTTCCTCACCAGGATGC		
ISG56-F	AGGAAACACCCACTTCGGTC	100	0.3 µM
ISG56-R	CCTCTAGGCTGCCCTTTTGT		
MAVS-F	CTGCCTCACAGCAAGAGACCA	181	0.3 µM
MAVS-R	GTAGACACAGGCCACTTCGTC		
GAPDH-F	GAAGGTGAAGGTCGGAGTCA	133	0.3 µM
GAPDH-R	CATGTAAACCATGTAGTTGAGGTC		
